# Optical Biometry-Based Intraocular Lens Calculation and Refractive Outcomes after Phacovitrectomy for Rhegmatogenous Retinal Detachment and Epiretinal Membrane

**DOI:** 10.1038/s41598-018-29553-w

**Published:** 2018-07-27

**Authors:** Nobuhiko Shiraki, Taku Wakabayashi, Hirokazu Sakaguchi, Kohji Nishida

**Affiliations:** Department of Ophthalmology, Osaka University Graduate School of Medicine, Suita, Osaka Japan

## Abstract

We investigate the refractive error after phacovitrectomy for rhegmatogenous retinal detachment (RRD; 100 eyes) and epiretinal membrane (ERM; 102 eyes). Axial lengths were measured by optical biometry in most patients. The main outcome measures were the refractive and absolute prediction errors. The overall mean refractive prediction error (ME) and mean absolute prediction error (MAE) were −0.40 ± 0.72 D and 0.62 ± 0.55 D, respectively, at 3 months postoperatively. The ME and MAE were significantly higher in the RRD group than in the ERM group (−0.63 ± 0.74 D vs −0.16 ± 0.63 D, *P* < 0.001 and 0.75 ± 0.62 D vs 0.49 ± 0.43 D, *P* = 0.002, respectively), indicating greater myopic shift in the RRD group. In the RRD group, adding +0.5 D to the preoperative predicted refractive power decreased the postoperative ME and MAE to −0.13 ± 0.74 D and 0.58 ± 0.47 D, respectively. Based on our results, we conclude that postoperative myopic shift was significantly higher in the RRD group than in the ERM group, possibly because of forward displacement of the intraocular lens by gas tamponade. The myopic shift can be minimized by adding +0.5 D to the predicted refractive power in patients undergoing phacovitrectomy for RRD.

## Introduction

Combined phacoemulsification, pars plana vitrectomy (PPV), and intraocular lens (IOL) implantation, known as phacovitrectomy, is now widely performed as a surgical treatment for vitreoretinal diseases in patients aged 50 years or older^1–5^. Advantages of combined phacovitrectomy over vitrectomy alone include faster visual recovery when compared with undergoing two separate procedures, safe vitreous shaving without concern for intraoperative lenticular touch or postoperative cataract progression^6–8^, and reduced the time and cost of surgery^9^. Recent advances in micro-incision vitrectomy surgery using small-gauge instrumentation (25-gauge or 27-gauge) and small incision (less than 2.2 mm) cataract surgery have further increased the safety and efficacy of combined phacovitrectomy^10–14^. However, there remains concern regarding a less predictable refractive outcome after phacovitrectomy^15–20^.

The IOL power calculation in combined phacovitrectomy has been performed similar to that in cataract surgery alone. In patients who undergo combined phacovitrectomy, deviations in refractive outcomes may be observed because of possible errors in measurement of axial length (AL), changes in the properties of the vitreous cavity after removal of the vitreous, or intraocular tamponade^15,19^. Most of the previous studies have reported variable degrees of myopic shift after phacovitrectomy for diabetic retinopathy, epiretinal membrane (ERM), and macular hole^15–19^. However, there have been few reports investigating the refractive outcomes after phacovitrectomy for rhegmatogenous retinal detachment (RRD)^20^.

To evaluate the degree of refractive error and further improve the refractive outcomes postoperatively, we compared the refractive outcomes after phacovitrectomy for RRD with those after phacovitrectomy for ERM in a relatively large group of patients.

## Results

Five hundred and ninety-five eyes of 595 consecutive patients with RRD or ERM were reviewed initially. Three hundred and ninety-three were excluded because of the exclusion criteria described earlier, leaving 202 eyes available for inclusion in the analysis. One hundred and two patients had ERM and 100 had RRD. In the patients with RRD, 61 (61%) had macula-on RRD and 39 (39%) had macula-off RRD. The patient characteristics are summarized in Table 1. The mean patient age was 64.8 ± 9.7 (range, 40–88) years. The AL measured by OB was used for the IOL power calculation in 187 (93%) eyes. The AL measured by A-scan ultrasonography was selected for the IOL power calculation in 15 eyes with RRD. The AL was significantly longer in patients with RRD (25.5 ± 1.7 mm) than those with ERM (24.3 ± 1.9 mm) (*P* < 0.001). Similarly, the number of eyes with AL greater than 26 mm was significantly higher in RRD (37 [37%] eyes) than in ERM (17 [17%] eyes) (*P* = 0.002). SF6 gas was used in all eyes in the patients with RRD but was not used at all in those with ERM. An IOL with a diameter of 7 mm was used more frequently in the patients with RRD than in those with ERM (*P* < 0.001). The overall mean preoperative logMAR BCVA was 0.22 ± 0.62. The mean logMAR BCVA was 0.13 ± 0.25 at 2 weeks and 0.43 ± 0.19 at 3 months after surgery. The mean BCVA improved significantly at 2 weeks (*P* = 0.02) and at 3 months (*P* < 0.0001) when compared with the preoperative BCVA.

**Table 1:** Clinical and demographic characteristics of the patients.

	Overall	RRD	ERM	*P*-value
Eyes/patients, n/n	202/202	100/100	102/102	
Age (y), mean ± SD	64.8 ± 9.7	61.7 ± 9.9	67.9 ± 8.4	<0.0001
Gender, n (%)				
Male	102 (51)	61 (61)	41 (40)	
Female	100 (49)	39 (39)	61 (60)	0.005
Preoperative BCVA				
Landolt C acuity chart, mean (range)	0.6 (HM–1.5)	0.58 (HM–1.5)	0.63 (0.1–1.5)	
LogMAR, mean ± SD	0.22 ± 0.62	0.24 ± 0.06	0.20 ± 0.06	0.66
Axial length				
Measured by optical biometry (mm), mean ± SD	24.9 ± 1.9	25.5 ± 1.7	24.3 ± 1.9	<0.0001
<26 mm, n (%)	148 (73)	63 (63)	85 (83)	
>26 mm, n (%)	54 (27)	37 (37)	17 (17)	0.002
Intravitreal SF_6_ tamponade during PPV, n (%)	100 (50)	100 (100)	0 (0)	<0.0001
IOL diameter used in surgery, n (%)				
6 mm	115 (57)	22 (22)	93 (91)	
7 mm	87 (43)	78 (78)	9 (9)	<0.0001

BCVA, best-corrected visual acuity; C_3_F_8_, perfluoropropane; ERM, epiretinal membrane; IOL, intraocular lens; logMAR, logarithm of the minimum angle of resolution; PPV, pars plana vitrectomy; RRD, rhegmatogenous retinal detachment; SD, standard deviation; SF_6_, sulfur hexafluoride.

### Refractive Outcome

In patients with RRD, the mean predicted refraction before surgery was −2.16 ± 1.85 D. The achieved postoperative refraction was −2.78 ± 2.08 D at 2 weeks and −2.8 ± 2.16 D at 3 months (Table 2). The difference between the predicted and achieved refraction was significantly different both at 2 weeks and at 3 months postoperatively (*P* < 0.001 and *P* < 0.001, respectively). In patients with ERM, the mean predicted refraction before surgery was −1.01 ± 1.13 D and the achieved postoperative refraction was −1.11 ± 1.33 D at 2 weeks and −1.18 ± 1.33 D at 3 months. The difference between the predicted and achieved refraction was not significantly different at 2 weeks but was significantly different at 3 months postoperatively (*P* = 0.07 and *P* = 0.006, respectively).

**Table 2:** Preoperative predicted and postoperative achieved refractive outcomes after phacovitrectomy for RRD and ERM.

Parameter	Predicted	Achieved	*P*-value
RRD			
2 weeks	−2.16 ± 1.85	−2.78 ± 2.08	<0.001
3 months	−2.16 ± 1.85	−2.80 ± 2.16	<0.001
ERM			
2 weeks	−1.01 ± 1.13	−1.11 ± 1.33	0.07
3 months	−1.01 ± 1.13	−1.18 ± 1.33	0.006

ERM, epiretinal membrane; RRD, rhegmatogenous retinal detachment.

In patients with RRD, the ME was −0.61 ± 0.73 D at 2 weeks and −0.63 ± 0.74 D at 3 months postoperatively (Table 3). The MAE was 0.72 ± 0.63 D at 2 weeks and 0.75 ± 0.62 D at 3 months postoperatively. In patients with ERM, the ME was −0.10 ± 0.66 D at 2 weeks and −0.16 ± 0.63 D at 3 months postoperatively. The MAE was 0.49 ± 0.45 D at 2 weeks and 0.49 ± 0.43 D at 3 months postoperatively. Therefore, both ME and MAE were significantly higher in the RRD group than in the ERM group (ME, *P* < 0.001 at 2 weeks, *P* < 0.001 at 3 months; MAE, *P* = 0.009 at 2 weeks, *P* = 0.002 at 3 months), indicating greater myopic shift in the RRD group. This tendency was observed in eyes with both preoperative AL > 26 mm and <26 mm (Table 3).

**Table 3:** Refractive outcomes

Parameter	Overall	RRD	ERM	*P*-value
ME (mean ± SD)				
2 weeks	−0.35 ± 0.74	−0.61 ± 0.73	−0.10 ± 0.66	<0.001
3 months	−0.40 ± 0.72	−0.63 ± 0.74	−0.16 ± 0.63	<0.001
MAE (mean ± SD)				
2 weeks	0.60 ± 0.55	0.72 ± 0.63	0.49 ± 0.45	0.009
3 months	0.62 ± 0.55	0.75 ± 0.62	0.49 ± 0.43	0.002
AL < 26 mm				
2 weeks				
ME	−0.36 ± 0.68	−0.63 ± 0.64	−0.15 ± 0.64	<0.001
MAE	0.58 ± 0.5	0.70 ± 0.56	0.5 ± 0.44	0.02
3 months				
ME	−0.38 ± 0.7	−0.62 ± 0.7	−0.2 ± 0.65	0.001
MAE	0.61 ± 0.51	0.74 ± 0.56	0.51 ± 0.45	0.01
AL > 26 mm				
2 weeks				
ME	−0.34 ± 0.89	−0.57 ± 0.88	0.18 ± 0.68	0.002
MAE	0.67 ± 0.68	0.75 ± 0.73	0.48 ± 0.50	0.15
3 months				
ME	−0.44 ± 0.79	−0.65 ± 0.81	0.03 ± 0.48	0.003
MAE	0.64 ± 0.63	0.75 ± 0.72	0.38 ± 0.27	0.08

AL, axial length; ERM, epiretinal membrane; MAE, mean absolute prediction error; ME, mean refractive prediction error; RRD, rhegmatogenous retinal detachment; SD, standard deviation.

In the RRD group, 44%, 68%, and 96% of eyes had a refractive prediction error within ±0.50 D, ±1.00 D, and ±2.00 D, respectively. In contrast, 65%, 90%, and 100% of eyes in the ERM group were within ±0.50 D, ±1.00 D, and ±2.00 D, respectively. The proportion of eyes with a predicted refractive error over ±0.5 D was significantly greater in the RRD group than in the ERM group (56% vs. 35%, *P* = 0.005).

### Factors Associated with Postoperative Refractive Error

Among the 202 eyes studied, univariate analysis revealed that type of disease (RRD or ERM) was significantly associated with postoperative refractive error at 3 months (*P* < 0.001). That is, patients with RRD had a significantly greater myopic shift than those with ERM (Table 4). Patient sex, preoperative BCVA, AL, keratometry (K) value, and IOL diameter (7 mm or 6 mm) were not associated with the postoperative refractive error at 3 months. In multivariate regression analysis, RRD was the only factor that was significantly associated with the postoperative refractive error at 3 months (*P* < 0.001).

**Table 4:** Univariate and multivariate regression analyses of the association between postoperative refractive error at 3 months and selected variables.

Variable	Univariate linear regression			Multivariate linear regression	
	Regression coefficient	R^2^	*P*-value	Regression coefficient	*P*-value
Patient sex (male, 0; female, 1)	−0.19	0.003	0.3		
Preoperative logMAR BCVA	0.11	0.01	0.06		
Axial length (mm)	−0.15	0.003	0.4		
K value	−0.04	0.009	0.17		
RRD or ERM (RRD, 1; ERM, 0)	1.1	0.09	<0.001	0.27	<0.001
IOL diameter (7 mm, 1; 6 mm, 0)	0.56	0.03	0.01		

BCVA, best-corrected visual acuity; ERM, epiretinal membrane; IOL, intraocular lens; logMAR, logarithm of the minimum angle of resolution; RRD, rhegmatogenous retinal detachment.

Sixty-one (61%) of the patients with RRD had macula-on RRD and 39 (39%) had macula-off RRD. There was no significant association between macular status and the outcomes with regard to postoperative refractive error.

### Refractive Outcomes with Correction Formula in RRD

If we added +0.5 D to the preoperative predicted refractive power, the postoperative ME and MAE decreased to −0.13 ± 0.74 D and 0.58 ± 0.47 D, respectively, in the RRD group (Table 5). The percentage of eyes with a predicted refractive error over ±0.5 D and ±1.0 D reduced to 46% and 16%, respectively, at 3 months.

**Table 5:** Refractive outcomes with correction formula at 3 months.

Parameter	Overall	RRD	ERM	*P*-value
ME (mean ± SD)	−0.15 ± 0.68	−0.13 ± 0.74	−0.16 ± 0.63	0.26
MAE (mean ± SD)	0.53 ± 0.45	0.58 ± 0.47	0.49 ± 0.43	0.08
AL < 26 mm				
ME (mean ± SD)	−0.17 ± 0.67	−0.12 ± 0.7	−0.2 ± 0.65	0.27
MAE (mean ± SD)	0.54 ± 0.43	0.58 ± 0.39	0.51 ± 0.45	0.1
AL > 26 mm				
ME (mean ± SD)	−0.1 ± 0.73	−0.15 ± 0.81	0.03 ± 0.48	0.78
MAE (mean ± SD)	0.52 ± 0.51	0.59 ± 0.72	0.38 ± 0.27	0.36

AL, axial length; ERM, epiretinal membrane; IOL, intraocular lens; logMAR, logarithm of the minimum angle of resolution; MAE, mean absolute prediction error; ME, mean refractive prediction error; RRD, rhegmatogenous retinal detachment.

## Discussion

In this study, we compared the refractive outcomes after phacovitrectomy for RRD and ERM in a relatively large number of patients. The mean refractive error was −0.63 ± 0.74 D in the RRD group and −0.16 ± 0.63 D in the ERM group 3 months after surgery. Therefore, the postoperative myopic shift was significantly greater in the RRD group than in the ERM group. In addition, multivariate regression analysis confirmed that type of disease (RRD), but not AL, preoperative BCVA, K value, or IOL diameter, was only the factor that was significantly associated with myopic shift postoperatively.

Several studies have reported that postoperative myopic shift occurs after phacovitrectomy because of the potential errors in AL measurement and forward movement of the IOL position caused by gas tamponade^15–19^. The AL has been measured by either A-scan ultrasonography or OB. OB determines the AL by measuring the distance from the tear film to the retinal pigment epithelium, and has been shown to be more accurate in measuring the AL, especially in eyes with retinal diseases^21^. Therefore, the AL was measured by OB in most patients in our study. AL was not associated with postoperative myopic shift, so we consider that AL was not incorrectly underestimated even in eyes with macula-off RRD. Indeed, the ME value did not differ significantly between macula-on and macula-off RRD. This result is consistent with a previous report that found no significant difference in refractive outcomes between macula-on and macula-off RRD^20^. Therefore, we speculate that the potential movement of the IOL position associated with gas tamponade, rather than the AL measurement, was the reason for the significant postoperative myopic shift in the eyes with RRD in our study.

The vitreous cavity was completely filled with SF6 gas at the end of surgery in all patients with RRD; however, SF6 gas tamponade was not performed in any of the patients with ERM. Although the patients in the RRD group maintained a face-down position in the early postoperative stage for retinal reattachment and prevention of forward displacement of the IOL, the SF6 gas may have forced the IOL forward and reduced the anterior chamber depth, resulting in a significant postoperative myopic shift. Schweitzer *et al*. reported a mean refractive error of −0.30 ± 0.66 D in 26 patients undergoing phacovitrectomy with gas tamponade for macular holes and 0.16 ± 0.55 D in 28 patients undergoing phacovitrectomy without gas tamponade^22^. Patel *et al*. also reported a mean refractive error of −0.39 ± 1.01 D in 40 eyes undergoing phacovitrectomy with gas tamponade for idiopathic macular holes, although they did not include a control group for comparison^17^. Further studies are needed to clarify the potential correlation between the degree of forward IOL displacement evaluated by anterior segment OCT and the myopic shift after phacovitrectomy with gas tamponade.

We expected that the larger IOL could prevent forward displacement of the IOL and associated myopic shift postoperatively. However, there was no significant association between ME values and IOL diameter (6 mm or 7 mm) in either the ERM group or the RRD group. Therefore, a larger IOL may not prevent the forward displacement of the IOL associated with gas tamponade.

The myopic shift of −0.63 D in patients with RRD undergoing phacovitrectomy with gas tamponade made us to consider modification of the IOL calculation to improve the postoperative refractive outcomes. To correct the myopic shift after phacovitrectomy in patients with RRD, we added +0.5 D to the preoperative predicted refractive power. The postoperative ME decreased to −0.13 ± 0.74 D. The percentage of eyes with a predicted refractive error over ±0.5 D and over ± 1.0 D decreased to 46% and 16%, respectively, at 3 months. Holladay *et al*. proposed that IOL calculation and refractive outcomes are favorable if 50% are within ±0.5 D and 90% are within ±1.0 D^23^. Therefore, we believe that adding +0.5 D to the preoperative predicted refractive power is acceptable to minimize the refractive error when the IOL is selected in patients with RRD.

The limitations of our present study include its retrospective design, a third generation formula but not a fourth or fifth generation formula used for IOL calculation because of the lack of AC depth data, and the lack of direct evidence of displacement of the IOL after phacovitrectomy with gas tamponade. Nevertheless, this study confirmed that the postoperative myopic shift was significantly greater in the RRD group than in the ERM group. Further studies may elucidate the potential correlation between degree of IOL displacement and myopic shift and the ocular biometric factors predictive of IOL displacement and myopic shift after phacovitrectomy.

In summary, the postoperative myopic shift was significantly higher in the RRD group. Adding +0.5 D to the preoperative predicted refractive power is recommended for better refractive outcomes in eyes with RRD.

## Methods

The study followed the tenets of the Declaration of Helsinki, and was approved by Ethics Committee of the Osaka University Graduate School of Medicine (10039). All patients provided written informed consent before surgery. Written informed consent was not considered to be necessary by the Ethics Committee to participate in this retrospective study.

We retrospectively undertook a chart review of consecutive patients who had undergone vitrectomy surgery for RRD or ERM between January 2014 and December 2015 at Osaka University Hospital. After the initial review, some eyes were excluded from the data analysis for the following reasons: sulcus fixation of the IOL, sulcus suture of the IOL, or intra-scleral IOL fixation because of intraoperative posterior capsule rupture; corneal disease, such as keratoconus, that interfered with refractive results; IOL implantation with a toric IOL or multifocal IOL; a follow-up period less than 3 months; a history of vitrectomy or corneal transplantation; other coexisting ocular disease such as schisis, macular hole, proliferative diabetic retinopathy, age-related macular degeneration, uveitis, acute retinal necrosis, Coat’s disease, proliferative vitreous retinopathy, or trauma; intraocular tamponade using silicon oil; and scleral buckling combined with vitrectomy. Preoperative data were collected, including a complete medical and ophthalmic history, preoperative refraction, AL, best-corrected visual acuity (BCVA), and characteristics of the RRD. The main outcome measures for data analysis were the refractive prediction error (i.e., postoperative actual refraction minus preoperative planned refraction) and absolute prediction error (i.e., absolute value of the difference between postoperative actual refraction and preoperative planned refraction).

### Preoperative Examinations and IOL Calculation

The AL was measured using optical biometry (OB; IOL Master; Carl Zeiss, Oberkochen, Germany). OB measures the AL from the tear film to the retinal pigment epithelium. If the AL measured by OB was shorter than that of the fellow eye, applanation A-scan ultrasonography (Nidek, Gamagori, Japan) was used to verify the AL measurement, as reported previously^20^. The IOL power was calculated using the SRK/T formula with the manufacturer’s recommended A-constant^24^. The axial length adjustment with Wang-Koch modification was not applied. The refractive value in the fellow eye determined the refractive aim in the operated eye.

All patients underwent ophthalmic examinations, including BCVA, refractive status, biomicroscopy of the anterior and posterior segments, indirect ophthalmoscopy, fundus photography, and spectral-domain optical coherence tomography (OCT; Cirrus HD-OCT 500; Carl Zeiss Meditec, Inc., Dublin, CA). The presence of ERM was detected by ophthalmoscopy and OCT. Macular status (on or off) in patients with RRD was confirmed by ophthalmoscopy or OCT before surgery.

### Surgical Procedure

Phacoemulsification was performed through a 2.4-mm or 2.2-mm clear corneal incision followed by 25-gauge or 27-gauge PPV using the Constellation® Vision System (Alcon Laboratories, Inc, Fort Worth, TX). The RESIGHT^™^ Fundus Viewing System (Carl Zeiss Meditec) or a contact lens system (Hoya Corporation, Tokyo, Japan) was used during PPV. Core vitrectomy and mid peripheral vitrectomy were performed followed by vitreous base shaving under scleral depression to remove the peripheral cortical gel. Perfluorocarbon liquid (Perfluoron®; Alcon Laboratories, Inc.) was used depending on the extent of the retinal detachment. An acrylic foldable IOL either 6 mm (one-piece) or 7 mm (3-piece) was implanted into the capsular bag. After IOL implantation, fluid-air exchange was performed and subretinal fluid was internally aspirated through the retinal breaks. Endolaser photocoagulation was applied using a curved laser probe around the retinal breaks. The vitreous cavity was replaced by a premixed nonexpansile concentration of 20% sulfur hexafluoride (SF6) by flushing with 50 ml syringe containing 20% SF6. Patients were instructed to remain face down for 3 to 8 days.

### Postoperative Examinations

Refraction was measured by an optometrist at 2 weeks and 3 months after surgery. Subjective refractions were used. The achieved postoperative refraction was expressed as a spherical equivalent. The main outcome measures were BCVA and the refractive prediction error, obtained by subtracting the achieved postoperative refraction at 3 months after the procedure from the predicted refraction before the procedure. The mean prediction error (ME) and mean absolute prediction error (MAE) were used as the refractive production error outcome measures^20^.

### Statistical Analysis

The statistical analysis was performed using JMP® version 11.2.0 statistical software (SAS Institute Inc., Cary, NC). Continuous values were expressed as the mean ± standard deviation. The visual acuity of each patient was converted to the logarithm of the minimum angle of resolution (logMAR) value for all calculations. Comparisons of continuous variables between subgroups were performed using the Wilcoxon rank sum test. Comparisons of continuous variables in one group were performed using the paired *t*-test. A *P*-value less than 0.05 was considered to be statistically significant.
